# New age constraints on the Lower Jurassic Pliensbachian–Toarcian Boundary at Chacay Melehue (Neuquén Basin, Argentina)

**DOI:** 10.1038/s41598-022-07886-x

**Published:** 2022-03-23

**Authors:** Aisha H. Al-Suwaidi, Micha Ruhl, Hugh C. Jenkyns, Susana E. Damborenea, Miguel O. Manceñido, Daniel J. Condon, Gladys N. Angelozzi, Sandra L. Kamo, Marisa Storm, Alberto C. Riccardi, Stephen P. Hesselbo

**Affiliations:** 1 Department of Earth Sciences, Khalifa University of Science and Technology, PO Box 12333, Abu Dhabi, UAE; 2 Department of Geology, Trinity College Dublin, The University of Dublin, Dublin 2, Ireland; 3 Department of Earth Sciences, University of Oxford, South Parks Road, Oxford, OX1 3AN UK; 4 División Paleozoología Invertebrados, Facultad de Ciencias Naturales y Museo, Universidad Nacional de La Plata, Argentina, CONICET, Paseo del Bosque S /N 1900, La Plata, Argentina; 5 British Geological Survey, Keyworth, NG12 5GG UK; 6 Laboratorio de Bioestratigrafía, Área de Geociencias, YPF Tecnología S.A., Baradero S/N, 1925, Ensenada, Argentina; 7 Jack Satterly Geochronology Laboratory, Department of Earth Sciences, University of Toronto, 22 Ursula Franklin St., Toronto, ON M5S 3B1 Canada; 8 Royal Netherlands Institute for Sea Research, Department of Marine Microbiology and Biogeochemistry, PO Box 59, 1790 AB Den Burg (Texel), The Netherlands; 9 Camborne School of Mines and Environment and Sustainability Institute, University of Exeter, Penryn Campus, Penryn, Cornwall, TR10 9FE UK

**Keywords:** Carbon cycle, Geochemistry, Geology, Sedimentology

## Abstract

The Pliensbachian–Toarcian boundary interval is characterized by a ~ 3‰ negative carbon-isotope excursion (CIE) in organic and inorganic marine and terrestrial archives from sections in Europe, such as Peniche (Portugal) and Hawsker Bottoms, Yorkshire (UK). A new high-resolution organic-carbon isotope record, illustrating the same chemostratigraphic feature, is presented from the Southern Hemisphere Arroyo Chacay Melehue section, Chos Malal, Argentina, corroborating the global significance of this disturbance to the carbon cycle. The negative carbon-isotope excursion, mercury and organic-matter enrichment are accompanied by high-resolution ammonite and nannofossil biostratigraphy together with U–Pb CA-ID-TIMS geochronology derived from intercalated volcanic ash beds. A new age of ~ 183.73 + 0.35/− 0.50 Ma for the Pliensbachian–Toarcian boundary, and 182.77 + 0.11/− 0.15 for the *tenuicostatum*–*serpentinum* zonal boundary, is assigned based on high-precision U–Pb zircon geochronology and a Bayesian Markov chain Monte Carlo (MCMC) stratigraphic age model.

## Introduction

The Early Jurassic Pliensbachian–Toarcian (Pl–To; ~ 184.2 Ma^1^) carbon-isotope excursion (CIE) is marked by a -3‰ shift in δ^13^C in both bulk-rock carbonate and organic carbon^2,3^. The stage boundary is associated in time with a second-order extinction event affecting ammonites, belemnites, gastropods, and many other benthic and pelagic groups, that effectively defines it^4–6^. This event precedes the onset of the Early Toarcian Oceanic Anoxic Event (T-OAE) and its associated Carbon Isotope Excursions (CIEs), appears relatively short-lived (~ 50–200 kyr^7^) and has been linked to the beginning of activity in the Karoo and Ferrar Large Igneous Provinces (LIP), recording an initial release of volcanogenic CO_2_ and other gases^8–10^.

The Pl–To event has been studied in the Tethyan and northwest European realms^2,7,11^, as well as Canada, Chile, and Japan^8,12–14^. The age of the boundary is presently computed based on a combination of cyclostratigraphy and U–Pb geochronology and is estimated to be 184.2 Ma based on an age of 183.2 ± 0.1 Ma for the top of the *tenuicostatum* Zone^1^. Other U–Pb ages that help to constrain this boundary include dates from Argentina^15^, Peru^16,17^, the USA^18^ and Canada^19–21^, although these lack tight biostratigraphic control, specifically with respect to correlation with the European ammonite zones. Dateable stratigraphic sections that can be bio- and chemostratigraphically correlated to marine sections elsewhere, specifically to the Global Stratotype Section and Point (GSSP) in Peniche, Portugal^6^, are essential to assigning a precise and accurate age to the base of the Toarcian. An improved age model for the Pl–To event offers greater insight into the driving mechanism of the observed environmental phenomena and the relationship with emplacement of the Karoo and Ferrar Large Igneous Provinces.

Here, we present a new high-resolution carbon-isotope chemostratigraphy and biostratigraphy that is calibrated using U–Pb ID-TIMS zircon dates for the Lower Jurassic (Pliensbachian–Toarcian) Chacay Melehue stream section in Neuquén Province, Argentina. A new age-depth model for this section is also presented, which constrains the age of both the Pliensbachian–Toarcian boundary and the onset of the negative carbon-isotope excursion in the earliest Toarcian. Using this new geochronology and biostratigraphy, combined with correlations to the GSSP and other well-defined sections, we explore the relationship between the Pliensbachian–Toarcian event and Karoo and Ferrar LIP activity.

## Palaeogeography and tectonic setting of the Neuquén Basin

**Figure 1:**
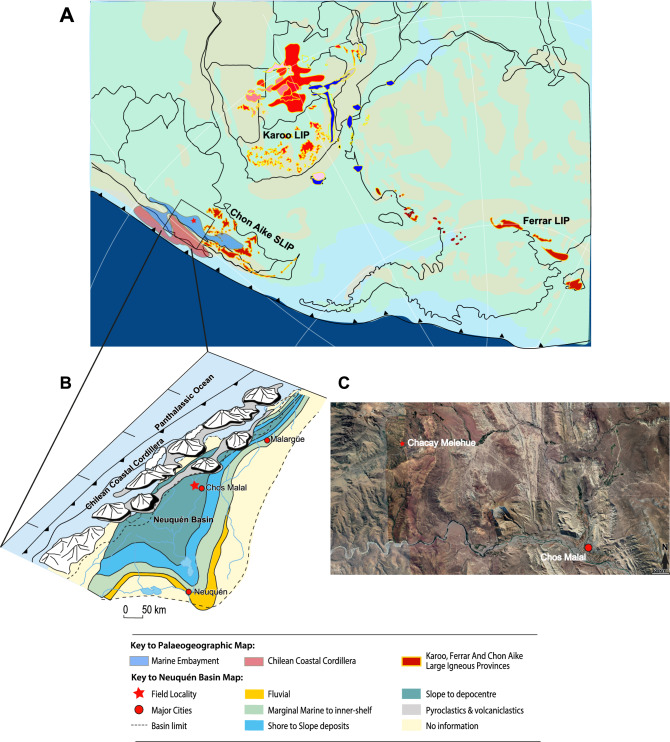
Palaeogeographic map for ∼ 183 Ma (after Blakey^22^) showing (**A**) the location of Karoo Ferrar Large mafic Igneous Province, the Chon Aike Silicic Igneous Province^23–27^, and the Neuquén Basin reconstruction (after Vicente^28^), and (**B**) the interior seaway, and depositional tracts within the basin (adapted and modified after^29–32^). Location of Chacay Melehue is indicated by red star. (**C**) Satellite image showing location of Chacay Melehue (red star, 37° 15′ 18.26ʺ S, 70° 30′ 16.34ʺ W,) relative to Chos Malal. (Satellite image courtesy of Google Earth Pro. 2020).

The Neuquén Basin is located on the eastern side of the Andes in west-central Argentina and central Chile, between 32° and 41° S (Fig. 1). The depositional area was a north–south-oriented back-arc basin and foreland, now containing more than 6 km of Triassic to Cenozoic sediments in its most central part^29^. The basin had a complicated tectonic history associated with the break-up of Gondwana, subduction of the proto-Pacific Plate and the development of the Andean magmatic arc^33^. Sediments were laid down in several depositional cycles representing deposition from the time of pre-rifting through to foreland-basin development^28^. The strata studied here form part of the marine Cuyo Group (Lower to Middle Jurassic). The deposition of the Cuyo Group was favoured by marine transgression during subsidence in the post-rift phase of basin development^33^. Sediments entered the Neuquén Basin from two main source areas: the Chilean Coastal Cordillera that supplied immature volcaniclastic material, and cratonic areas to the south and northeast from which more mineralogically mature sediment was derived^34–36^.

## Chacay Melehue stratigraphy and depositional setting

The Arroyo Chacay Melehue stratigraphic section presented here is located at S37°15′ 18.15ʺ, W70°30′ 26.55ʺ (Fig. 1) and comprises more than ~ 1200 m of sediment spanning the latest Pliensbachian to Oxfordian interval^37,38^. At the base of the section are epiclastic and pyroclastic deposits of the La Primavera Formation, which are thought to have been derived from an andesitic strato-volcano complex, referred to as the Chilean Coastal Cordillera, on the western side of the Neuquén embayment during the latest Triassic–Early Jurassic^39,40^ (Fig. 1).

**Figure 2:**
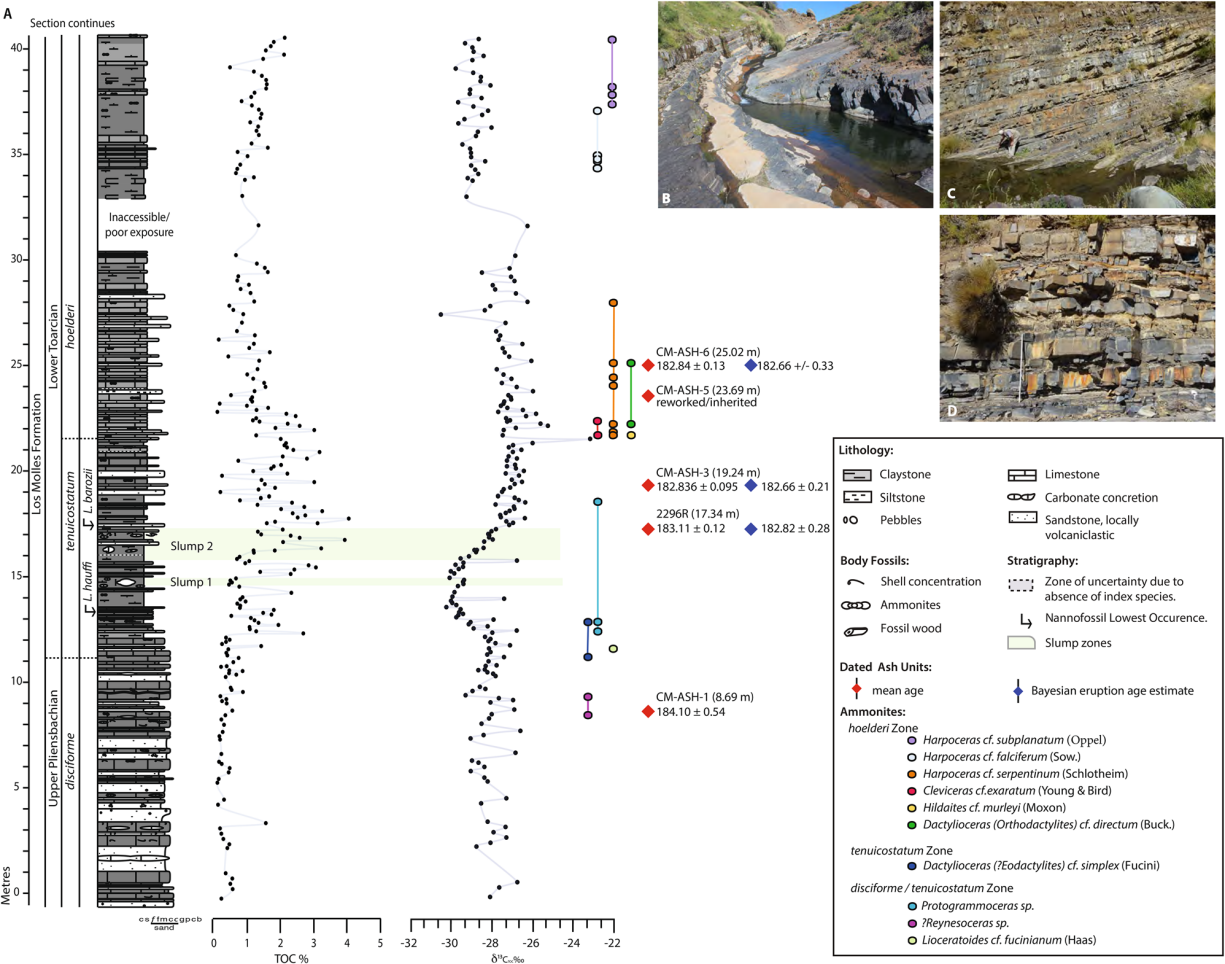
(**A**) Carbon-isotope chemostratigraphy including main formation names, lithology, total organic carbon (TOC) content, ammonite zones and occurrences, nannofossil biostratigraphy and radiometric ages for the Pliensbachian–Toarcian transition in the Chacay Melehue stream section. Photographs of Chacay Melehue Section: (**B**) light-coloured tuffaceous units interbedded in dark grey lithified units (~ 3 m in section), (**C**) unit with nodular base, thin tuffaceous units and well-indurated dark grey mudstones (~ 14 m to 18 m in section), and (**D**) sediments typical for the upper part of the section, with thin interbedded tuffaceous units (> 20 m in the section).

Previous studies of sedimentary units at Chacay Melehue suggest that the section was deposited in a marginal marine to offshore environment, recording transgressive–regressive cycles of sedimentation within the Neuquén Basin^41^. Tuffaceous beds present throughout the section are typically fining upwards and inferred to be largely fine-grained turbidites, redepositing previously laid down ash beds. The presence of discrete volcaniclastic beds at the bottom of the section, and the presence of volcaniclastic material in the sandstone beds throughout, indicates that the section was proximal to a volcanic arc situated to the west^42^ (Fig. 2). Up-section, coarser grained material decreases in relative abundance, suggesting that either the grain size from the source area changed or that the basin experienced a relative sea-level rise, increasing the distance between source and depocentre at Chacay Melehue. A deepening environment is also suggested by the presence of dark-coloured shale units with organic enrichment stratigraphically above 11 m in the section, suggesting deposition in an oxygen-depleted environment (Fig. 2).

The presence of two distinct, slumped deposits (14.5–17 m, Fig. 2) may suggest increased weathering and local sediment overloading at Pl–To boundary time, possibly due to an enhanced hydrological cycle^43^. Percival et al.^44^ and Xu et al.^35^ have previously suggested enhanced continental weathering during the Pl–To boundary interval and T-OAE based on excursions in Os^187^/Os^188^, as well as evidence of centimetre-scale gravity-flow deposits from the T-OAE interval in the Mochras core, Cardigan Bay Basin, UK. Many other records of the T-OAE/CIE also show similar evidence for an enhanced hydrological cycle and increased weathering and erosion during this event, coinciding with and/or following Karoo and Ferrar volcanism^12,46^.

## Geochronological and biostratigraphic constraints at Chacay Melehue

Ammonites and other fossils were sampled wherever found in situ, and tuffaceous samples were collected throughout the section (full details of horizons and determinations are given in the supplementary data).

Biostratigraphic determination of the Chacay Melehue section confirms the presence of deposits of Late Pliensbachian through earliest Toarcian age (Fig. 2). This section was previously studied for geochronology^15,47^. Sample 2296R collected at 17.34 m in the Chacay Melehue section (see supplementary Fig. 1), and located in the *tenuicostatum* zone ~ 6 m above the Pliensbachian–Toarcian boundary, was analysed by Riccardi & Kamo^15^. This sample has a mean ^206^Pb/^238^U age of 183.11 ± 0.12 and a Bayesian eruption age estimate^48^ of 182.82 ± 0.28 Ma.

Here, we have analysed 4 additional samples, CM-ASH-1, 3, 5 and 6, from within the same section. Data are corrected to the EARTHTIME tracer ET535, based on U–Pb CA-ID-TIMS analyses of individually abraded zircon crystals (see supplementary information section for details on the methodology).

CM-ASH-1, at 8.69 m, has an estimated maximum depositional age of 184.10 ± 0.54 Ma and sits in the latest Pliensbachian *disciforme* Andean ammonite zone, equivalent to the latest *margaritatus*–*spinatum* northwest European ammonite zones^50–52^. The bivalve *Kolymonectes weaveri* Damborenea is also present here from 0.50 to 12.74 m in the section and has an established stratigraphic range from the Late Pliensbachian through the Early Toarcian^53^. CM-ASH-1 occurs ~ 5 m below the lowest and first occurrence (FO) of the nannofossil *Lotharingius hauffii* Grün & Zwili (FO 13.55 m), which has an age range from the Late Pliensbachian, NJ5a subzone to the Callovian, NJ12a subzone^54^.

CM-ASH-3, at 19.24 m, gives a mean age of 182.836 ± 0.0951 Ma and a Bayesian eruption age estimate of 183.66 ± 0.21 Ma. CM-ASH-3 is located above the FO of *Lotharingius barozii* Noël (at 17.34 m; ash 2296R occurs at the same level). *Lotharingius barozii* Noël is characteristic of the latest Pliensbachian to earliest Toarcian *disciforme–tenuicostatum* Andean ammonite zones^50,55,56^ as well as occurring above the FO of *Dactylioceras (Eodactylites)* cf. simplex (Fucini, FO 11.08 m) indicative of the early Toarcian *tenuicostatum* Zone^57^. The base of the Andean *hoelderi* Zone is identified in the section at 21.66 m and is marked by the FO of *Harpoceras serpentinum* (Schlotheim), *Cleviceras exaratum* (Young & Bird) and *Hildaites* cf*. murleyi* (Moxon). The Andean *hoelderi* Zone is considered approximately equivalent to the *serpentinum* (= *falciferum*) ammonite Zone of northwestern Europe^52,55^.

At the GSSP for the base of the Toarcian at Peniche (Portugal), the FO of *Lotharingius barozii* is in strata of the uppermost *emaciatum* ammonite Zone, ~ 50 cm below the base of the Toarcian Stage. Furthermore, the Pliensbachian–Toarcian boundary at this locality is marked by the FO of *Dactylioceras (Eodactylites) simplex,* which is considered to allow global correlation of this level, thereby providing strong support for the proposition that the geochronology in this part of the Chacay Melehue section constrains the age of the boundary. CM-ASH-5 at 23.68 m did not yield an interpretable age as the zircons are inherited or reworked.

The final ash dated in this study, CM-ASH-6 at 25.02 m, gives a weighted mean U–Pb date of 182.84 ± 0.13 Ma, and a Bayesian eruption age estimate of 183.66 ± 0.33 Ma^48^ and is located ~ 4.5 m above the FO of *Harpoceras serpentinum* (Schlotheim), *Cleviceras exaratum* (Young & Bird) and *Hildaites* cf. *murleyi* (Moxon) (FO 21.66 m) within the Andean *hoelderi* Zone, equivalent to the *serpentinum* (= *falciferum*) ammonite Zone of northwestern Europe^52,55,57^.

Leanza et al.^47^ also sampled and analyzed two ash beds in the Chacay Melehue locality using U–Pb CA-ID-TIMS: one of the ashes, at ~ 24 m in the section, yielded an age of 185.7 ± 0.40 Ma; this bed is located biostratigraphically above the Pliensbachian–Toarcian boundary, is cross-bedded, and has a very wide array of zircon ages within the zircon population. Consequently, it appears likely that the bed is largely made up of reworked volcaniclastic material, despite the tightly clustered age ranges of the youngest zircons that contribute to this precise date, but probably do not give an accurate depositional age. A second ash bed was dated by Leanza et al.^47^, which produced an age of 182.3 ± 0.4 Ma; its exact stratigraphic position within the succession is, however, unknown with respect to our measured section. Field photographs in Leanza et al.^47^ could not be matched to the outcrop at the times of our field investigations.

**Figure 3:**
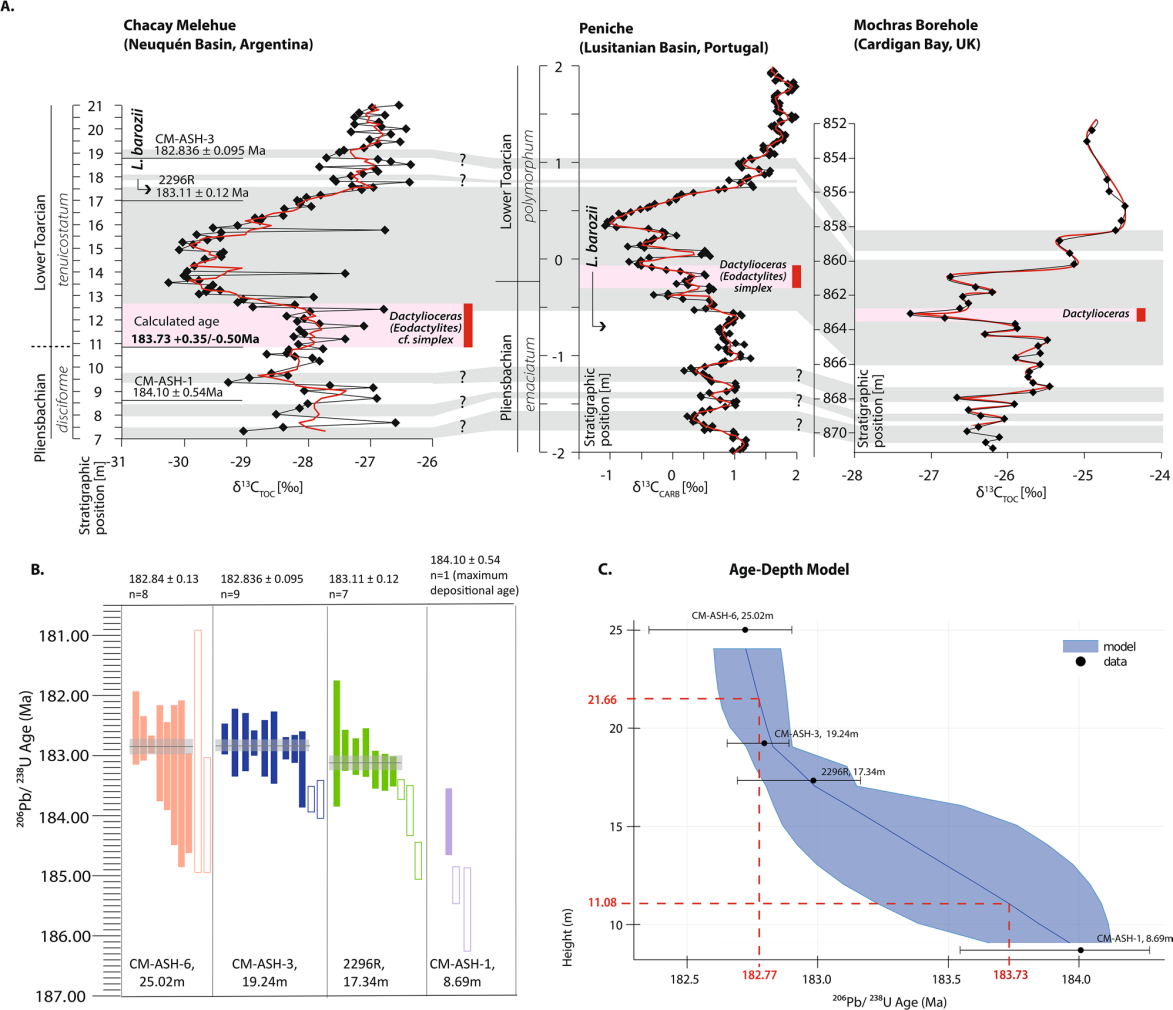
(**A**) Detailed comparison of Chacay Melehue geochronology, carbon-isotope chemostratigraphy and the lowest and first occurrence (FO) of *Dactylioceras* (indicated by the horizontal pink band and vertical red bar) as compared with the GSSP at Peniche, Portugal^3,6^ and in the Mochras borehole, UK^58^. Grey bands highlight chemostratigraphic correlation intervals between the two localities. Pink bands highlight the occurrence of key ammonites of latest Pliensbachian and Early Toarcian age in the sections. Red line shows 2 pt. moving average (**B**) Shows the distribution of zircons from individual ash bed units in the Chacay Melehue stratigraphic section. Open rectangles indicate ages considered detrital and not used to determine the mean age or in the Age-Depth model. (**C**) Age–depth model for Chacay Melehue showing the Bayesian distribution of the ages and the modelled age estimates. The red dashed line and emboldened red numbers indicate the modelled age for the Pliensbachian–Toarcian boundary at 11.08 m, with a modelled age of ~ 183.73 + 0.35/-0.50 Ma, and for the *tennuicostatum*–*hoelderi* zonal boundary (equivalent to the *tenuicostatum*–*serpentinum* zonal boundary) at 21.66 m, with a modelled age of ~ 182.77 + 0.11/-0.15 Ma.

To improve constraints on the age of the Pliensbachian–Toarcian boundary and the age of the lower Toarcian *tenuicostatum–hoelderi* boundary we used Chron.jl^49^, which is a model framework that allows the interpretation of mineral age spectra in a stratigraphic context. Chron.jl^48^ uses a Bayesian Markov chain Monte Carlo (MCMC) model in which stratigraphic superposition is imposed on U–Pb zircon dates^49^. The result is an age–depth model incorporating dates from all beds above and below each sample to produce an internally consistent age (Fig. 3B,C.). This model allowed us to extrapolate ages at specific depths, assuming relatively constant sedimentation rates of the deposits between the ash beds that provide the geochronological constraints (Fig. 3C). To determine the age of the Pliensbachian–Toarcian boundary, we assessed the stratigraphic position of the boundary to be at 11.08 m in the section, concurrent with the FO of *Dactylioceras (Eodactylites),* and interpolated the age to be 183.73 + 0.35/− 0.50 Ma (Fig. 3). A similar exercise was performed for the *tenuicostatum–hoelderi* zone boundary (concurrent with the *tenuicostatum–serpentinum* zone boundary in NW Europe), using the FO of *Harpoceras serpentinum* (Schlotheim), *Cleviceras exaratum* (Young & Bird) and *Hildaites* cf. *murleyi* (Moxon) (FO 21.66 m). Thus, at 21.66 m in the section an age of 182.77 + 0.11/− 0.15 Ma was interpolated from the model (Fig. 3C).

The age–depth model coupled with biostratigraphy provides a new more precise age for two of the major events in the earliest Toarcian as well as a new age for the Pliensbachian–Toarcian boundary.

## The Pliensbachian–Toarcian boundary carbon-isotope excursion

Total organic carbon (TOC) concentrations across the studied stratigraphic interval range from values of 0–1% in the uppermost Pliensbachian *disciforme* Zone (0 to 11 m, Fig. 2), to values of 1.5–4% in the *tenuicostatum* Zone (11 to 22 m), and values of 0.5–1% higher up in the section. As the TOC content increases up through the *tenuicostatum* Zone, the δ^13^C_TOC_ record shows a marked negative shift, initiated at ~ 13 m in the studied section (Fig. 3), and with values gradually falling from a background of ~ − 27.5‰, to − 30.1‰ at ~ 15 m (Fig. 3). The δ^13^C_TOC_ values above ~ 16 m in the section shows a gradual positive shift, returning to ~ − 26.5‰ at ~ 18 m. Subsequently, from ~ 18 to 30 m in the section, δ^13^C_TOC_ values are relatively stable, oscillating by 1–2‰ around an average value of − 27‰ (Fig. 2). In the upper part of the studied section, above a poorly exposed stratigraphic interval, δ^13^C_TOC_ values are significantly more negative, averaging around ~ − 29‰ and falling as low as − 29.8‰; this shift to lower values coincides with a gradual increase to relatively more elevated TOC values of up to ~ 2% in this uppermost part of the section. *T*_max_ °C values range from 296 to 506 °C throughout the section, Hydrogen Index values range from 3 to 23 mg HC/gTOC, and S2/S3 < 1 (S2 = mg hydrocarbons/ g rock, S3 = mg CO_2_ / rock; RockEval data are available in supplementary data file), suggesting that organic matter in the section is made up of higher plant material and/or hydrogen-poor organic constituents that have been oxidized and/or suffered thermal maturation^59^.

**Figure 4:**
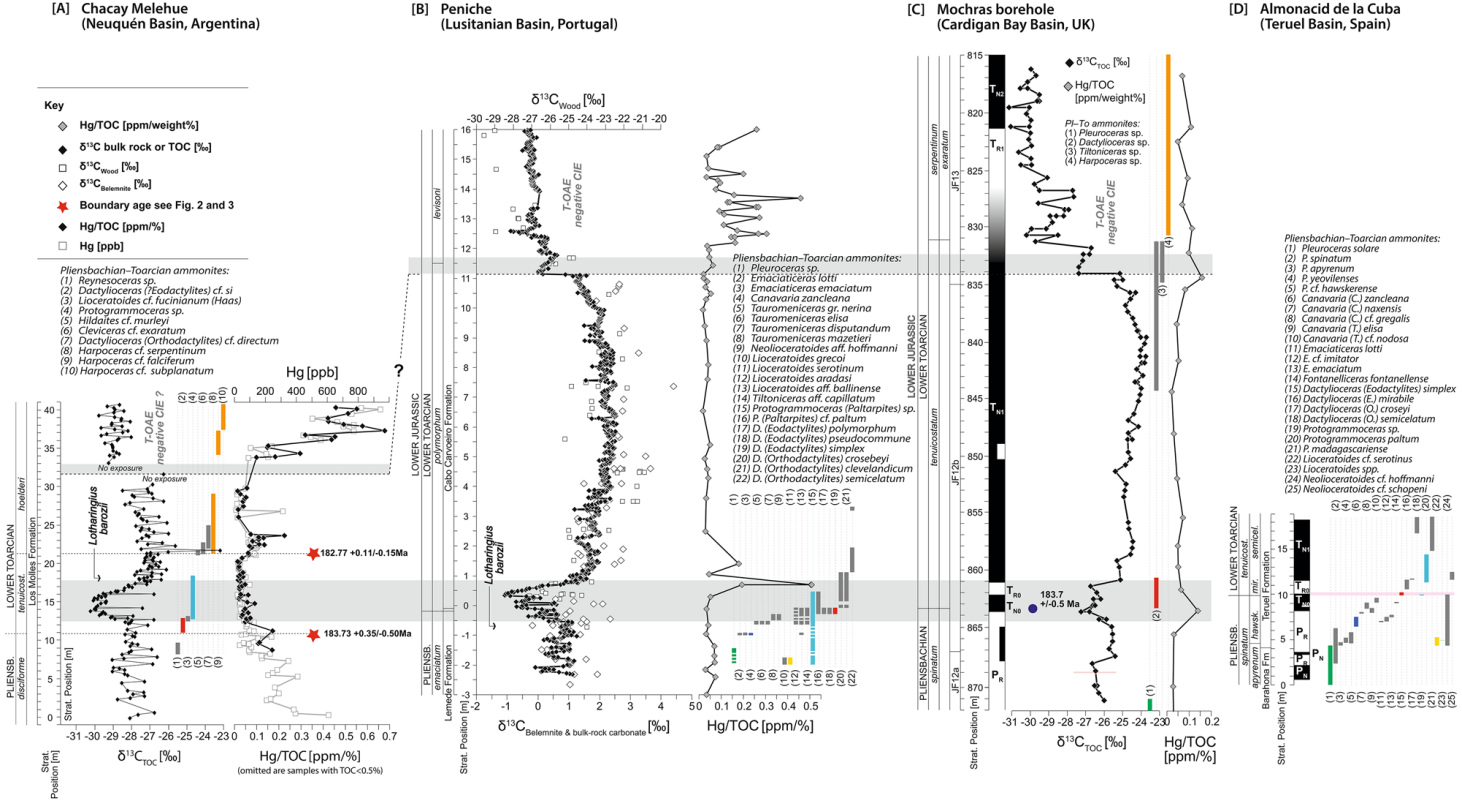
Comparison of Hg/TOC, [Hg], carbon isotopes, magnetostratigraphy and ammonite biostratigraphy from Chacay Melehue (this study), Peniche (Lusitanian Basin, Portugal)^2,6^, the Mochras Borehole including astronomical age of 183.70 ± 0.50^60^ (Cardigan Bay Basin, UK)^45,58,60,61^, and the Almonacid de la Cuba section (Spain)^62,63^. Grey bands indicate the Pliensbachian–Toarcian boundary and the Toarcian carbon-isotope excursion. Key ages calculated in this study are also indicated by red stars.

The carbon-isotope profile of Chacay Melehue can be chemostratigraphically correlated to other biostratigraphically well-constrained sections, specifically to the base-Toarcian GSSP in Peniche, Portugal^6^ (Fig. 3). The δ^13^C signatures of Chacay Melehue (bulk organic carbon) and Peniche (bulk carbonate) show a remarkably similar ~ 2‰ negative carbon-isotope excursion across the Pl–To boundary. Additionally, the combined chemo-, chrono- and biostratigraphic framework from Chacay Meleue is here also compared and correlated with other stratigraphically well-constrained sections such as from the Mochras borehole, Cardigan Bay Basin, UK^45,60,61^ and Almonacid de la Cuba, Teruel Basin, Spain^51^ (Fig. 4, Supp. Fig. 2).

In the Chacay Melehue section, sedimentary mercury [Hg] concentrations are 300–700 ppb in the lowest 5 m of the section with values decreasing to 20–50 ppb through the sediments displaying the negative excursion in the section (~ 10 to 20 m; Fig. 4). Hg/TOC values show a small increase at the Pl–To transition, against a falling trend and, at around 23 m in the studied succession, with values of up to 0.23 ppm/wt%, are followed upwards by reduced values of ~ 0.05 ppm/weight% (Fig. 4). Hg/TOC values strongly increase up to 0.67 ppm/weight % towards the top of the studied succession, coinciding also with increasing TOC values and decreasing δ^13^C_TOC_ values (Fig. 4), possibly representing the onset of the T-OAE negative CIE. The observed trend in the Hg/TOC profile at Chacay Melehue is similar in shape and order of magnitude to other records, such as at Mochras (Cardigan Bay Basin, UK) and Peniche (Lusitanian Basin, Portugal^61^; Fig. 4).

## Age implications of Chacay Melehue chemo-, chrono- and biostratigraphy for the Pliensbachian–Toarcian boundary and T-OAE

**Figure 5:**
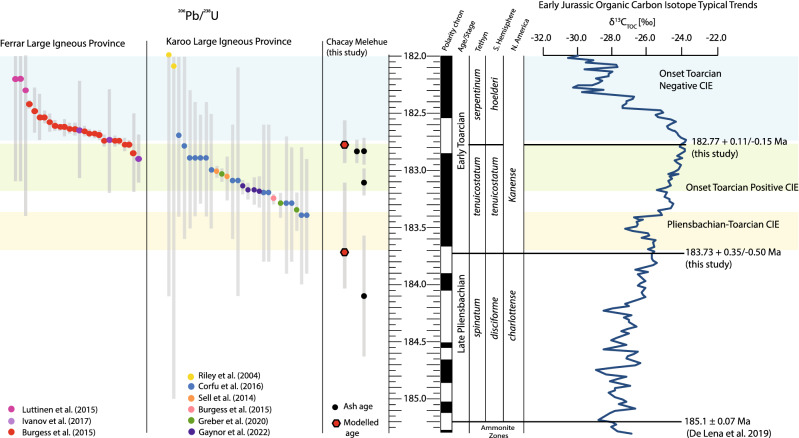
Distribution of ^206^Pb/^238^U absolute ages from the Karoo and Ferrar Large Igneous Provinces, and dates from this study on a numerical timescale, relative to the carbon-isotope data (2-pt moving average) from the Mochras Borehole (Cardigan Bay Basin, UK^45,60^), spanning the Pliensbachian–Toarcian transition. The numerical timescale is obtained using age tie-points for the ammonite zone boundaries based on geochronological constraints from this study, and linear interpolation in between. Major carbon-cycle perturbations are also indicated. Magnetic polarity scale adapted from Hesselbo et al.^1^ (References for Karoo and Ferrar dates are available in the supplementary data file).

The onset of environmental perturbations at the Pl–To boundary likely resulted in global warming, oceanic anoxia, intensified weathering, and a calcification crisis, in a similar manner to, and setting the stage for, the larger perturbations recorded during the Toarcian Oceanic Anoxic Event that had its focus in the *serpentinum* Zone (= ~ *falcifererum* Zone =  ~ *hoelderi* Zone). Caruthers et al.^8,64^ suggested that the long-term environmental change that resulted in pulsed extinction events in the Pliensbachian–Toarcian appear to have been associated with the onset and peaks of intrusive magmatism in Karoo, Ferrar and silicic volcanism in Chon Aike (Figs. 1, 5); however, these igneous provinces are chemically distinct, and resulted in different environmental impacts. For example, the Karoo LIP was emplaced relatively rapidly and intruded into Permian organic-rich sediments^65–68^ (Fig. 5), whereas Chon Aike, which is a silicic Large Igneous Province, was emplaced over a longer period and likely did not result in rapid hydrothermal venting of greenhouse gases, but more gradual gaseous release over a relatively long period from ~ 160–190 Ma^69^.

The chemostratigraphy from Chacay Melehue strengthens the case for the global nature of the previously observed Pl–To negative carbon-isotope excursion and disturbance to the carbon cycle. The ~ 3‰ negative excursion in δ^13^C_TOC_ values closely follows the stratigraphically lowest occurrence of *Dactylioceras (Eodactylites)* cf. *simplex* (Fucini) in the section, a taxon closely allied to the principal marker for the base Toarcian GSSP at Peniche, Portugal^6^ (Fig. 4).

In addition, the Chacay Melehue section provides new constraints for the age of the Pl–To boundary at ~ 183.73 + 0.35/− 0.50 Ma, as well as for the *tenuicostatum–serpentinum* zonal boundary at ~ 182.77 + 0.11/− 0.15 Ma, with the latter occurring stratigraphically close to the onset of the negative carbon-isotope excursion associated with the T-OAE.

These dates and zonal durations are consistent with recent astrochronological estimates for the ages of this boundary^60^, which suggest a million-year duration for the earliest Toarcian *tenuicostatum* (or concurrent *polymorphum*) Zone^7,60,70–73^. Furthermore, astrochronological constraints on the duration of the Pl–To negative CIE suggest a duration of ~ 200 kyr^7,72^, which agrees with the geochronological constraints on the duration of this event, as illustrated here.

Integrated global correlation of the Chacay Melehue data with other successions well documented by ammonite biostratigraphy, chemostratigraphy, magnetostratigraphy and/or geochronology (Fig. 4), demonstrate that the Pl–To boundary event may be tied to the onset of LIP activity in Karoo but pre-dates the peak of substantial magmatism in Karoo and Ferrar by ~ 400 kyr (Fig. 5). This relationship between the Pl-To boundary event and the onset of Karoo magmatic activity is further supported by the increase in elemental mercury in the Chacay Melehue section, and correlative records (Fig. 4), inferred to have been volcanogenically derived and transported through the atmosphere before final deposition in marine sediments.

## Supplementary Information

Supplementary Information 1.

Supplementary Figure 1.

Supplementary Figure 2.
