# Supplementary material for: New age constraints on the Lower Jurassic Pliensbachian–Toarcian Boundary at Chacay Melehue (Neuquén Basin, Argentina)
Source: Sci Rep. 2022 Mar 23;12:4975. doi: 10.1038/s41598-022-07886-x (PMC8942990; doi:10.1038/s41598-022-07886-x)

Supplementary Figure 1

Chacay Melehue, Neuquén, Argentina

Hesselbo notebook 30.3.08 – 2.4.08

Samples: Al-Suwaidi, Damborenea, Hesselbo, Jenkyns, Manceñido

Supplementary figure showing detailed stratigraphic log, sample locations, ammonite, bivalve and nanofossil determinations.

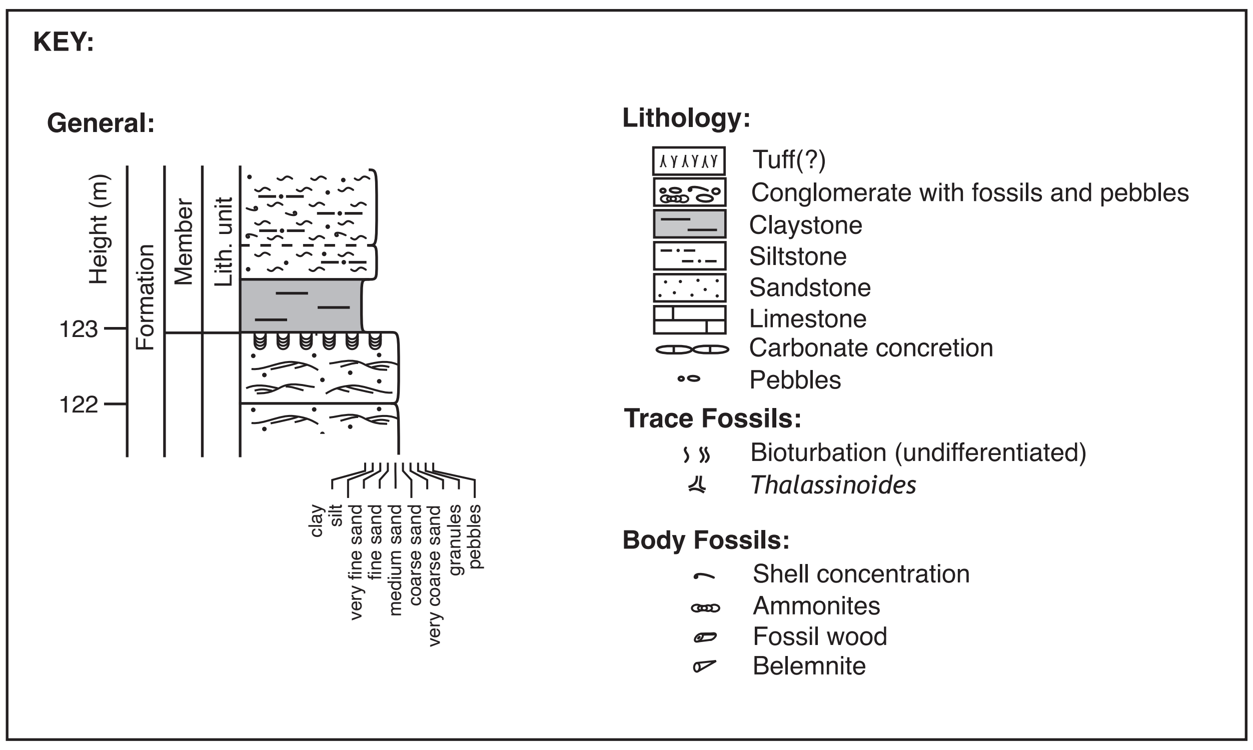

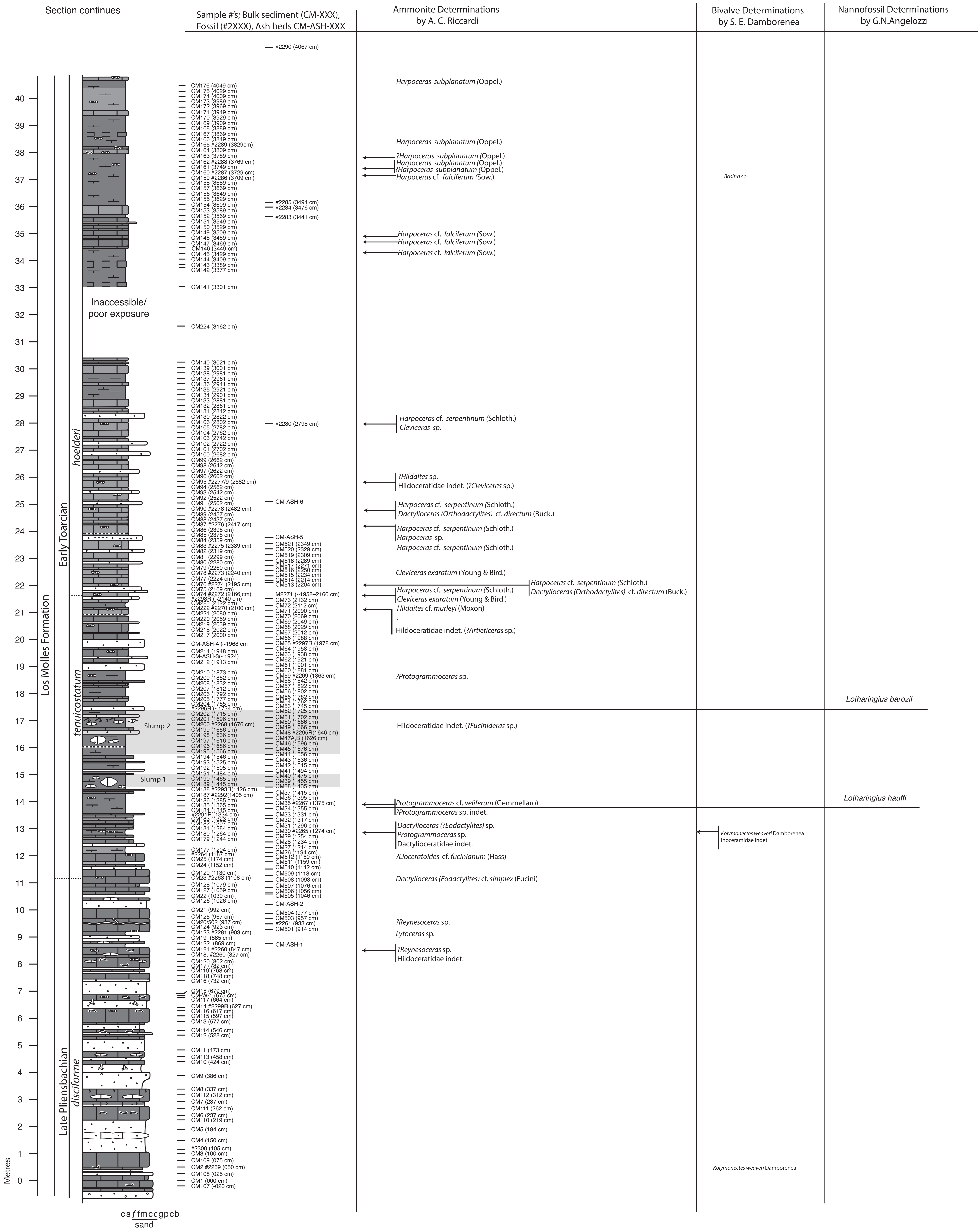

Supplement: Supplementary file 2 — Supplementary Figure 1. [file 41598_2022_7886_MOESM2_ESM.pdf]
