# Supplementary material for: New age constraints on the Lower Jurassic Pliensbachian–Toarcian Boundary at Chacay Melehue (Neuquén Basin, Argentina)
Source: Sci Rep. 2022 Mar 23;12:4975. doi: 10.1038/s41598-022-07886-x (PMC8942990; doi:10.1038/s41598-022-07886-x)
Supplement: Supplementary file 3 — Supplementary Figure 2. [file 41598_2022_7886_MOESM3_ESM.pdf]

Supplementary Figure 2. Comparative Carbon Isotopes for Chacay Melehue and Mocharas Core

Carbon isotope chemostratigraphy for Chacay Melehue and the Mochras core (compiled from Xu et al. (2019) and Storm et al. (2020)). The figure shows details of key ammonites, carbon isotopes and calculated and absolute ages for the two sections. This data is highlighted here as a supplementary file as the generalised Pliensbachian–Toarcian carbon isotope curve in Figure 5 is generated by integrating the age constraints from Chacay Melehue as well as ammonite biostratigraphy and the very high resolution carbon isotopes in the Mochras core to generate a time controlled carbon isotope curve.

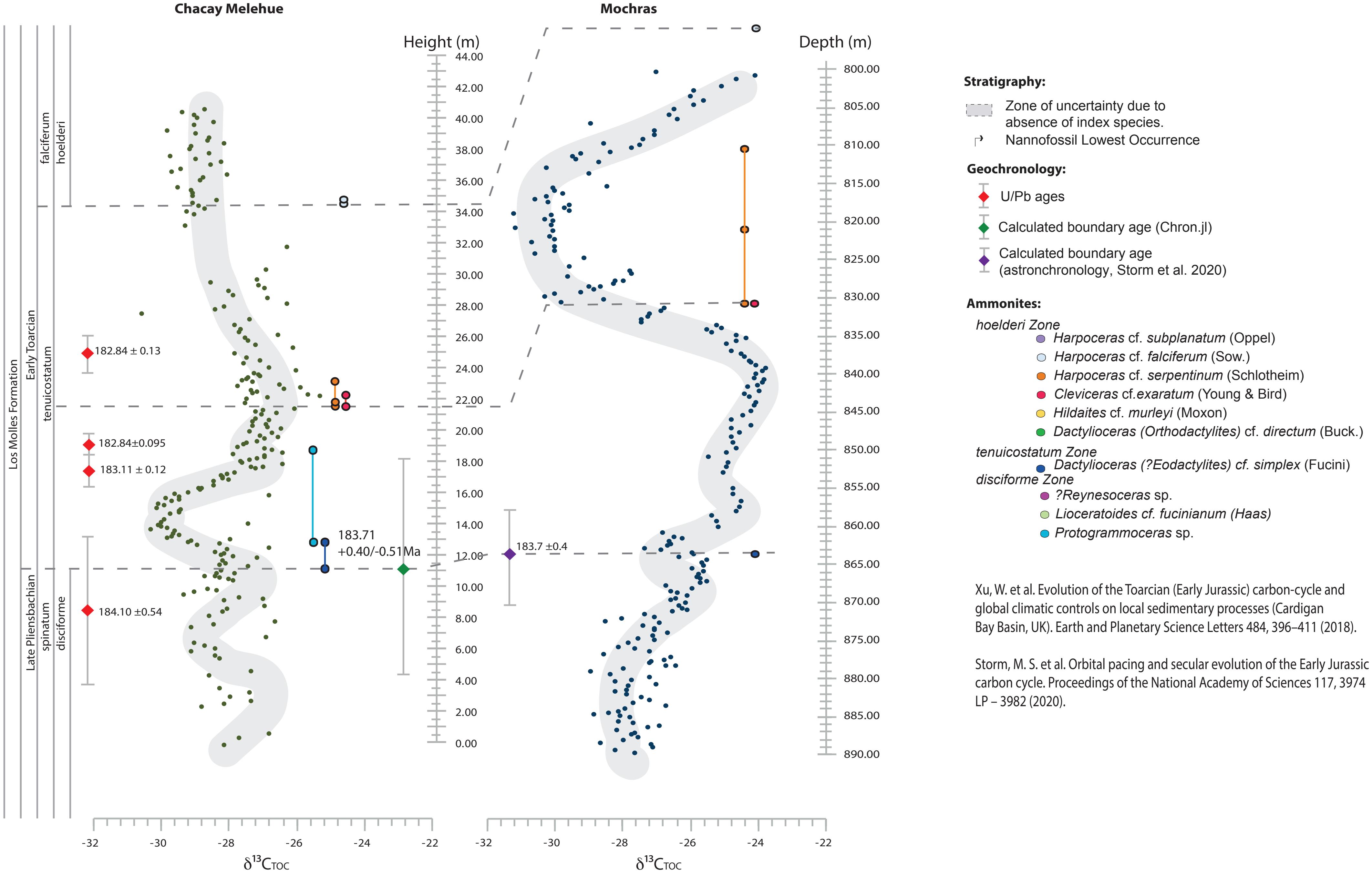

Xu, W. et al. Evolution of the Toarcian (Early Jurassic) carbon-cycle and global climatic controls on local sedimentary processes (Cardigan Bay Basin, UK). Earth and Planetary Science Letters 484, 396–411 (2018).

Storm, M. S. et al. Orbital pacing and secular evolution of the Early Jurassic carbon cycle. Proceedings of the National Academy of Sciences 117, 3974 LP – 3982 (2020).
